# Safety and efficacy of apatinib in combination with chemotherapy with or without immunotherapy versus chemotherapy alone as first-line treatment for advanced gastric cancer

**DOI:** 10.1007/s10637-024-01423-5

**Published:** 2024-02-17

**Authors:** Lele Chang, Xuemei Zhang, Qian Ma, Lingyang Kong, Yang Yu, Ji Tao, Qingwei Li

**Affiliations:** 1 Department of Gastrointestinal Medical Oncology, Harbin Medical University Cancer Hospital, 150 Haping Road, Harbin, Heilongjiang Province 150001 China; 2 Department of Radiation Oncology, Quzhou People’s Hospital, Quzhou, China; 3 Cancer Center, The First Hospital of Jilin University, Changchun, China

**Keywords:** Stomach neoplasms, Apatinib, Immunotherapy, Chemotherapy, Efficacy

## Abstract

The specific first-line regimen for advanced gastric cancer (GC) is still controversial. The benefit of apatinib for first-line treatment of advanced GC remains unknown and needs to be further explored. Eighty-two patients with advanced GC treated in our institution from October 2017 to March 2023 were retrospectively reviewed. All individuals had her-2 negative GC and had received at least two cycles of first-line treatment, including 44 patients in the combination treatment group (apatinib in combination with chemotherapy with or without immunotherapy) and 38 patients in the simple chemotherapy group. We evaluated the efficacy and safety of apatinib in combination with chemotherapy with or without immunotherapy in the first-line treatment of advanced GC by comparing the efficacy, progression-free survival (PFS), and adverse events in two groups of patients. The median PFS of the simple chemotherapy group was 9.25 months (95% confidence interval (CI), 6.1–11.2 months), and that of the combination treatment group was 10.9 months (95% CI, 7.9–15.8 months), which was 1.65 months longer than the simple chemotherapy group. Statistically significant differences are shown (*P* = 0.022). The objective response rate (ORR) of the combination treatment group was 65.9%, and 36.8% in the simple chemotherapy group. Statistically significant differences are shown (*P* = 0.014). No serious (Grade IV) adverse events occurred in either group. Our study indicates that apatinib in combination with chemotherapy with or without immunotherapy as first-line treatment for advanced GC exhibits good anti-tumor activity and is well tolerated by patients.

## Introduction

Gastric cancer (GC) is one of the leading causes of cancer-related fatalities [1]. According to statistics, more than 70% of GC patients come from Southeast Asia, and China accounts for the majority [2]. Since early GC usually has no obvious clinical symptoms, most GC patients are diagnosed when they have localized invasion or distant metastases [1, 3]. Advanced GC loses the opportunity for radical surgery, and the 5-year survival rate is < 20% [3]. Comprehensive treatment based on chemotherapy (CT) prolongs the survival of GC patients [4]. Although CT can improve the survival rate of patients, most patients will eventually develop recurrence or drug resistance, leading to disease progression [5–7]. Therefore, there is an urgent need for safer and more effective treatment options. 

One of the important processes in the malignant growth of tumors is angiogenesis. Sustained angiogenesis is crucial for tumor growth and invasion, and vascular endothelial growth factor (VEGF) and vascular endothelial growth factor receptor (VEGFR) play essential roles in tumor angiogenesis progression [8–10]. Apatinib is a new generation of small-molecule tyrosine kinase inhibitors; its main target is VEGFR2 [11]. In 2014, this drug was approved in China for late-line treatment of patients with advanced GC [12, 13]. However, despite the use of anti-angiogenic drugs, the median overall survival (OS) of individuals with advanced GC is less than 2 years [14, 15].

With the continuous development of immunotherapy, the combined use of immune checkpoint inhibitors (ICI) and anti-angiogenic drugs has shown better anti-tumor effects [16–22]. ICI has been approved for second-line and later-line treatment of individuals with microsatellite instability-high (MSI-H) and different mismatch repair (dMMR) advanced GC [16–18]. Currently, several clinical studies are trying to study the inhibitory effect of ICI on tumors in the first-line or neoadjuvant treatment phase [23–25]. With the advance of treatment lines, the OS of advanced GC patients continues to be prolonged.

Apatinib and ICI have showcased excellent results in the late-line therapy of advanced GC, but reliable evidence for their use in first-line treatment is currently lacking. Hence, we undertook a retrospective study to explore the efficacy of apatinib in combination with chemotherapy with or without immunotherapy in the first-line treatment of advanced GC patients and to provide new ideas and theoretical basis for clinical treatment.

## Materials and methods

### Patient selection

This research enrolled 82 individuals with advanced GC who were treated at the Harbin Medical University Cancer Hospital from October 2017 to March 2023. The main inclusion criteria are as follows: (1) pathologically diagnosed as GC by two professional pathologists, and her-2 negative or her-2 status is unknown; (2) the patient’s bone marrow reserve function and liver function are grossly normal, and the estimated survival time is ≥ 180 days; (3) advanced or postoperative recurrence GC patients with measurable target lesions; (4) eastern cooperative oncology group (ECOG) score, 0–2 points; (5) all of the enrolled individuals were treated as first-line treatment, had received at least 2 cycles of systemic therapy, and had received at least one clinical evaluation of the efficacy; (6) the patient’s tumor TNM stage is III to IV. Exclusion criteria include the following: (1) second primary malignancies were reported in the past 5 years; (2) uncontrolled hypertension; (3) no measurable target lesions, lack of or incomplete clinical data, incomplete follow-up information, etc.

This research was approved by the Ethics Committee of the Harbin Medical University Cancer Hospital (approval number: KY2023-18) and was conducted in accordance with the Declaration of Helsinki. All individuals sign written informed consent.

### Treatment strategies

Combined treatment group (apatinib in combination with chemotherapy with or without immunotherapy), patients received oral apatinib (500 mg once daily) on days 12–21. Immunotherapy includes the following: sintilimab, 200 mg, day 1; or toripalimab, 240 mg, day 1; or camrelizumab, 200 mg, day 1. Chemotherapy regimens include the following: S-1 single-agent regimen, 40–60 mg/m^2^, twice daily, days 1–14; or SOX (oxaliplatin + S-1) regimen, oxaliplatin, 130 mg/m^2^, days 1–14; S-1 as above; or nab-paclitaxel + S-1 regimen, nab-paclitaxel, 125 mg/m^2^, days 1 and 8; S-1 as above; or XELOX (oxaliplatin + capecitabine) regimen, oxaliplatin, 130 mg/m^2^, day 1 and capecitabine, 1000 mg/m^2^, days 1–14. Each cycle lasts 21 days. The simple chemotherapy group contains only one chemotherapy regimen, without other anti-tumor treatments. If significant adverse events (AEs) occur, dose reduction may be required. Treatment continued until disease progression, unacceptable toxicity, or any other causes. Patients in either group had regular blood routine monitoring, liver function, renal function, coagulation function, and other indicators.

### Clinical data

We collected the patient’s gender, age, ECOG score, histologic differentiation, primary tumor location, previous gastrectomy, carbohydrate antigen 199 (CA199) and carcinoma embryonic antigen (CEA) value before the first first-line treatment, metastasis information (peritoneal metastasis, number of metastatic lesions), chemotherapy regimen, and chemotherapy cycles. Follow-up data and efficacy evaluation information were extracted from our institution’s electronic medical record system and telephone contacts. The last follow-up time will be on November 1, 2023.

### Efficacy and safety

The primary endpoint is progression-free survival (PFS), and secondary endpoints are disease control rate (DCR), ORR, OS, and safety. PFS is the time from the first treatment to the patient’s progression or death, and OS (Since the last follow-up was on November 1, 2023, OS has not been reached in some patients, which will be reported in a subsequent article.) is the time from the first treatment to any cause of death. Patients in both groups were evaluated for efficacy using Response Evaluation Criteria in Solid Tumors (RECIST 1.1), including complete response (CR), partial response (PR), stable disease (SD), and progressive disease (PD). The ORR was defined as the ratio of the sum of CR plus PR. The DCR was defined as the ratio of the sum of CR, PR, and SD. We use the common terminology criteria for adverse events (CTCAE) standard version 5.0 which classifies AEs.

### Statistical analysis

Data analysis was performed using SPSS V.26.0 software (IBM Corp) and R (Version 4.3.1). Report continuous measurement data that follows a normal distribution is the mean (standard deviation), and those do not obey the normal distribution are presented as the median (first quartile, third quartile). Categorical variables were expressed as the number of cases (percentage), and the *χ*2 test or Fisher test was used for comparison between groups. Survival curves were constructed using the Kaplan–Meier method and log‐rank test was used for comparison. COX proportional hazard analysis was used to conduct univariate and multivariate analysis to explore the impact of variables on survival rate. *P* < 0.05 indicated statistical significance.

## Result

### Clinicopathological characteristics of patients

Table 1 shows baseline patient characteristics. Patients were classified into the combined treatment group and the simple chemotherapy group according to whether used apatinib with or without immunotherapy. The combined treatment group had 44 patients, and the simple chemotherapy group included 38 patients. The ECOG score range of all patients was 0–2. No significant differences were observed between the two groups in terms of differences in gender, age, ECOG score, tumor location, previous gastrectomy, CEA, CA199, metastasis status (peritoneal metastasis, number of metastatic lesions), and chemotherapy cycles.

**Table 1:** Baseline clinical characteristics of the two groups of study population

Variables	Simple chemotherapy group	Combined treatment group	*P*‐value
	(*N* = 38)	(*N* = 44)	
Gender			0.643
Female	11 (28.9%)	15 (34.1%)	
Male	27 (71.1%)	29 (65.9%)	
Age	59.4 (11.1)	55.8 (11.3)	0.146
ECOG			0.067
0	19 (50.0%)	31 (70.5%)	
1	17 (44.7%)	9 (20.5%)	
2	2 (5.26%)	4 (9.09%)	
Histologic differentiation			0.033
Well	1 (2.63%)	0 (0.00%)	
Moderately	0 (0.00%)	7 (15.87%)	
Poorly	19 (50.0%)	18 (40.9%)	
Unknown	18 (47.4%)	19 (43.2%)	
Tumor location			0.553
Esophagogastric junction	3 (7.89%)	4 (9.09%)	
Fundus of stomach	1 (2.63%)	1 (2.27%)	
Body of stomach	9 (23.7%)	8 (18.2%)	
Antrum	21 (55.3%)	19 (43.2%)	
Pylorus	1 (2.63%)	1 (2.27%)	
Lesser curvature of stomach	2 (5.26%)	5 (11.4%)	
Greater curvature	1 (2.63%)	6 (13.6%)	
Previous gastrectomy			0.116
No	37 (97.4%)	38 (86.4%)	
Yes	1(2.6%)	6 (13.6%)	
CA199	19.6 [9.99, 76.5]	23.0 [6.31, 105]	0.978
CEA	4.90 [2.00, 28.1]	3.31 [1.46, 9.01]	0.245
Peritoneal metastasis			0.816
No	26 (68.4%)	28 (63.6%)	
Yes	12 (31.6%)	16 (36.4%)	
Number of metastatic lesions			0.191
≤ 2	15 (39.5%)	24 (54.5%)	
> 2	23 (60.5%)	20 (45.5%)	
Chemotherapy regimens			0.020
S-1	1 (2.63%)	0 (0.00%)	
SOX	24 (63.2%)	39 (88.6%)	
Nab-paclitaxel + S-1	4 (10.5%)	3 (6.82%)	
XELOX	9 (23.7%)	2 (4.55%)	
Chemotherapy cycles	6.00 [4.00, 8.00]	5.00 [3.75, 7.00]	0.075
ORR	14 (36.8%)	29 (65.9%)	0.014
DCR	26 (68.5%)	37 (84.1%)	0.119

*ECOG* Eastern Cooperative Oncology Group, *CA199* carbohydrate antigen 199, *CEA* carcinoembryonic antigen, *S-1* tegafur–gimeracil–oteracil potassium, *SOX* S-1 plus oxaliplatin, *XELOX* capecitabine plus oxaliplatin, *ORR* objective response rate, *DCR* disease control rate

### Patient short-term outcomes and survival analysis

**Fig. 1:**
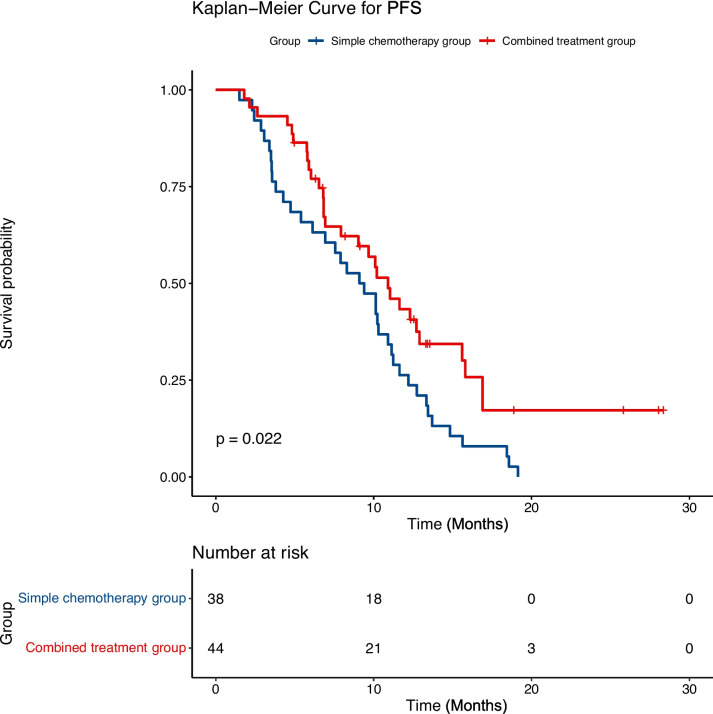
Kaplan–Meier curves of progression‐free survival (PFS) between combined treatment group and simple chemotherapy group

**Fig. 2:**
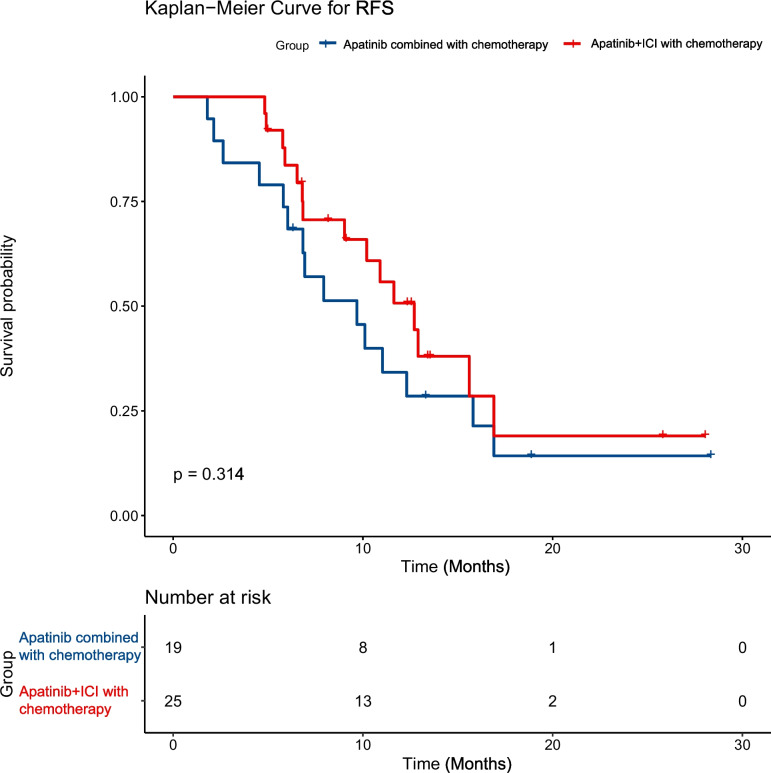
Kaplan–Meier curves of progression‐free survival (PFS) between apatinib plus ICI with chemotherapy group and apatinib combined with chemotherapy group

The ORR rate of the combined treatment group (65.9%) was significantly better than that of the simple chemotherapy group (36.8%) (*P* = 0.014). Although the DCR of the combined treatment group was 84.1%, which was better than the 68.5% of the simple chemotherapy group, no significant difference between these two groups was found (*P* = 0.119) (Table 1). The survival curve demonstrated that the PFS of the combined treatment group was substantially longer than that of the simple chemotherapy group (10.9 months; 95% CI, 7.9–15.8 vs. 9.25 months 95% CI, 6.1–11.2; *P* = 0.022, Fig. 1). In the subgroup analysis of the combined treatment group, although the PFS of the apatinib combined with ICI with chemotherapy group was prolonged versus the apatinib combined with chemotherapy group, it did not show statistical significance (12.7 months; 95% CI, 9.0-NA vs. 9.68 months, 95% CI, 6.8–16.9; *P* = 0.314, Fig. 2). As of the last follow-up time (November 1, 2023), among the 19 patients in the apatinib combined with chemotherapy group, 12 (63.1%) patients had PR, 2 (10.5%) patients had PD, the DCR was 89.4%, and the ORR was 63.1%. Among the 25 patients in the apatinib combined with ICI with chemotherapy group, 17 (68.0%) patients showed PR, 5 (20%) patients developed PD, the ORR was 68.0%, and the DCR was 80.0%. Both groups of patients showed better ORR and DCR, but no statistically significant differences between the two groups were observed (Table 2).

**Table 2:** Treatment efficacy and survival condition for the combined treatment group

Variables, *n* (%)	Apatinib combined with chemotherapy group	Apatinib + ICI with chemotherapy group	*P*‐value
	(*N* = 19)	(*N* = 25)	
CR	0 (0%)	0 (0%)	0.398
PR	12 (63.1%)	17 (68.0%)	
SD	5 (26.3%)	3 (12.0%)	
PD	2 (10.5%)	5 (20.0%)	
ORR	12 (63.1%)	17 (68.0%)	0.759
DCR	17 (89.4%)	20 (80.0%)	0.680

*ICI, immune checkpoint inhibitors, CR* complete response, *PR* partial response, *SD* stable disease, *PD* progressive disease, *ORR* objective response rate, *DCR* disease control rate

### Univariate and multifactorial results of clinicopathological features associated with RFS

Univariate analysis of all 82 patients showed that whether peritoneal metastasis occurred, different chemotherapy regimens, chemotherapy cycles, and combined treatment group were related to RFS (*P* < 0.05, Table 3). Then, multivariate COX regression analysis was conducted on variables with *P* < 0.05. The results demonstrated that peritoneal metastasis (*P* < 0.001), XELOX chemotherapy regimen (*P* = 0.0291), chemotherapy cycle ≥ 6 cycles (*P* < 0.001), and combined treatment group (*P* < 0.001) are independent prognostic factors for PFS (Table 3).

**Table 3:** Univariate and multivariate Cox regression analysis of PFS prognostic factors

Variables	Univariate analysis		*P*‐value	Multivariate analysis		*P*‐value
	HR	95% CI		HR	95% CI	
Gender						
Female	1	Reference				
Male	0.68	(0.41–1.13)	0.139			
Age						
< 60	1	Reference				
≥ 60	0.75	(0.46–1.21)	0.243			
ECOG						
0	1	Reference				
1	0.99	(0.59–1.65)	0.965			
2	1.67	(0.65–4.29)	0.283			
Histologic differentiation						
Well	1	Reference				
Moderately	1.36	(0.16–11.38)	0.775			
Poorly	1.45	(0.2–10.74)	0.715			
Unknown	1.7	(0.23–12.57)	0.602			
Tumor location						
Esophagogastric junction	1	Reference				
Fundus of stomach	0.91	(0.11–7.85)	0.931			
Body of stomach	1.91	(0.68–5.38)	0.219			
Antrum	1.4	(0.55–3.58)	0.48			
Pylorus	2.38	(0.45–12.58)	0.308			
Lesser curvature of stomach	3.02	(0.94–9.71)	0.063			
Greater curvature	0.46	(0.11–1.95)	0.294			
Previous gastrectomy						
No	1	Reference				
Yes	0.52	(0.19–1.44)	0.208			
CA199						
≤ 20	1	Reference				
> 20	1.24	(0.76–2.01)	0.391			
CEA						
≤ 4	1	Reference				
> 4	0.97	(0.6–1.57)	0.894			
Peritoneal metastasis						
No	1	Reference		1	Reference	
Yes	2.19	(1.34–3.6)	0.002	3.05	(1.79–5.19)	< 0.001
Number of metastatic lesions						
≤ 2	1	Reference				
> 2	1.03	(0.64–1.66)	0.899			
Chemotherapy regimens						
S-1	1	Reference		1	Reference	
SOX	0.13	(0.02–1.01)	0.052	0.19	(0.02–1.54)	0.1197
Nab-paclitaxel + S-1	0.24	(0.03–2.08)	0.195	0.36	(0.04–3.27)	0.3635
XELOX	0.08	(0.01–0.67)	0.02	0.08	(0.01–0.78)	0.0291
Chemotherapy cycles						
< 6	1	Reference		1	Reference	
≥ 6	0.46	(0.28–0.74)	0.002	0.36	(0.21–0.62)	< 0.001
Treatment						
Simple chemotherapy group	1	Reference		1	Reference	
Combined treatment group	0.58	(0.36–0.93)	0.024	0.36	(0.21–0.62)	< 0.001

*HR* hazard ratio, *95% CI*, 95% confidence interval, *ECOG* Eastern Cooperative Oncology Group, *CA199* carbohydrate antigen 199, *CEA* carcinoembryonic antigen, *S-1* tegafur–gimeracil–oteracil potassium, *SOX* S-1 plus oxaliplatin, *XELOX* capecitabine plus oxaliplatin

### Adverse events (AEs)

All 82 patients completed at least two cycles of treatment. Table 4 summarizes the AEs for enrolled patients. Overall, the most common hematological AEs in the simple chemotherapy group and combined treatment group were leukopenia (63.2% vs. 84.1%), while the most common non-hematological adverse events were vomiting and loss of appetite (78.9% vs. 95.5%). No serious (Grade IV) AEs occurred in patients in both groups. No serious (G3–G5) immune-related adverse reactions (iRAEs) occurred in the 25 patients in the apatinib combined with ICI with chemotherapy group. Only a few patients experienced immune-related rash, immune fever, and other adverse reactions. The symptoms were mild and well tolerated. They continued to use ICI drugs without interruption.

**Table 4:** Adverse drug reactions for all patients

Complication, *n* (%)	Simple chemotherapy group (*N* = 38)				Combined treatment group (*N* = 44)				Total (*N* = 82)			
	I	II	III	Total	I	II	III	Total	I	II	III	Total
**Hematological**												
Leukopenia	17 (44.7%)	6 (15.8%)	1 (2.6%)	24 (63.2%)	21 (47.7%)	12 (27.3%)	4 (9.1%)	37 (84.1%)	38 (46.3%)	18 (22.0%)	5 (6.1%)	61 (74.4%)
Neutropenia	12 (31.6%)	6 (15.8%)	1 (2.6%)	19 (50%)	15 (34.1%)	8 (18.2%)	2 (4.5%)	25 (56.8%)	27 (32.9%)	14 (17.1%)	3 (3.7%)	44 (53.7%)
Anemia	15 (39.5%)	5 (13.2%)	3 (7.9%)	23 (60.5%)	19 (43.2%)	7 (15.9%)	3 (6.8%)	29 (65.9%)	34 (41.5%)	12 (14.6%)	6 (7.3%)	52 (63.4%)
Thrombocytopenia	7 (18.4%)	4 (10.5%)	1 (2.6%)	1 2(31.6%)	13 (29.5%)	5 (11.4%)	2 (4.5%)	20 (45.5%)	20 (24.4%)	9 (11.0%)	3 (3.7%)	32 (39.0%)
**Non‐hematological**												
Vomiting or loss of appetite	19 (50.0%)	9 (23.7%)	2 (5.3%)	30 (78.9%)	28 (63.6%)	10 (22.7%)	4 (9.1%)	42 (95.5%)	47 (57.3%)	19 (23.2%)	6 (7.3%)	72 (87.8%)
Proteinuria	14 (36.8%)	4 (10.5%)	1 (2.6%)	19 (50%)	18 (40.9%)	4 (9.1%)	2 (4.5%)	24 (54.5%)	32 (39.0%)	8 (9.8%)	3 (3.7%)	43 (52.4%)
Hypoproteinemia	9 (23.7%)	4 (10.5%)	2 (5.3%)	15 (39.5%)	14 (31.8%)	5 (11.4%)	2 (4.5%)	21 (47.7%)	22 (26.8%)	8 (9.8%)	2 (2.4%)	32 (39.0%)
Elevated transaminases	10 (26.3%)	4 (10.5%)	1 (2.6%)	15 (39.5%)	12 (27.3%)	4 (9.1%)	1 (2.3%)	17 (38.6%)	23 (28.0%)	9 (11.0%)	4 (4.9%)	36 (43.9%)
Hyperbilirubinemia	9 (23.7%)	2 (5.3%)	1 (2.6%)	12 (31.6%)	9 (20.5%)	2 (4.5%)	1 (2.3%)	12 (27.3%)	18 (22.0%)	4 (4.9%)	2 (2.4%)	24 (29.3%)
Sensory neuropathy	13 (34.2%)	5 (13.2%)	2 (5.3%)	20 (52.6%)	16 (36.4%)	5 (11.4%)	2 (4.5%)	23 (52.3%)	29 (35.4%)	10 (12.2%)	4 (4.9%)	43 (52.4%)
Abdominal pain	8 (21.1%)	1 (2.6%)	1 (2.6%)	10 (26.3%)	10 (22.7%)	2 (4.5%)	1 (2.3%)	13 (29.5%)	18 (22.0%)	3 (3.7%)	2 (2.4%)	23 (28.0%)
Diarrhea	8 (21.1%)	2 (5.3%)	1 (2.6%)	11 (28.9%)	12 (27.3%)	2 (4.5%)	1 (2.3%)	15 (34.1%)	20 (24.4%)	4 (4.9%)	2 (2.4%)	26 (31.7%)
Hypertension	11 (28.9%)	4 (10.5%)	1 (2.6%)	16 (42.1%)	10 (22.7%)	6 (13.6%)	3 (6.8%)	19 (43.2%)	21 (25.6%)	10 (12.2%)	4 (4.9%)	35 (42.7%)
Hand-foot syndrome	12 (31.6%)	4 (10.5%)	1 (2.6%)	17 (44.7%)	15 (34.1%)	4 (9.1%)	1 (2.3%)	20 (45.5%)	27 (32.9%)	8 (9.8%)	2 (2.4%)	37 (45.1%)

## Discussion

Our findings indicated that the median PFS was prolonged by approximately 1.65 months in the combination therapy group (10.9 months; 95% CI, 7.9–15.8 months), which was superior to that of the simple chemotherapy group (9.25 months; 95% CI, 6.1–11.2 months; *P* = 0.022). Compared with the simple chemotherapy group, the ORR of patients in the combination treatment group increased by 29.1% (65.9% vs. 36.8%, *P* = 0.014), and the DCR increased by 15.6% (84.1% vs. 68.5%, *P* = 0.119). These results indicated that advanced GC patients seem to confer benefit from apatinib in combination with chemotherapy with or without immunotherapy as first-line treatment. Compared with the apatinib combined with chemotherapy group, the ORR of the patients in the apatinib combined with ICI with chemotherapy group was increased, the PFS was prolonged, and the DCR was decreased, but no statistical differences were observed. This may be due to the small sample of patients and insufficient patient follow-up time. Unfortunately, OS events are not yet mature in this study, the long-term survival rate and OS rate are unclear, and we will further update our study results later. Since 2014, apatinib has been approved by China for late-line treatment of advanced GC patients [12, 13]. Researchers are exploring it in depth, from third-line to second-line treatment and then from second-line treatment to first-line treatment and neoadjuvant treatment. Apatinib has shown good therapeutic effects. However, the results of an exploratory, single-arm, phase II trial [26] showed that the PFS of apatinib plus S-1 as first-line therapy of advanced GC was only 4.21 months (95% CI, 2.29–6.13 months) and did not outperform other chemotherapy regimens, which may be because only a single chemotherapy drug is used and cannot control tumor growth. Peng et al. [27] conducted a study of camrelizumab combined with CAPOX and then camrelizumab combined with apatinib maintenance as the first-line treatment of advanced GC, and the findings indicated that the ORR of this combination regimen was 58.3%, PFS was 6.8 months (95% CI, 5.6–9.5 months). Our findings suggested that the PFS of the combination treatment group was 10.9 months, 95% CI, 7.9–15.8 months, which may be due to the joint effect of apatinib in combination with chemotherapy with or without immunotherapy regimen. In addition, some research has suggested that apatinib also plays a crucial role in the neoadjuvant treatment of GC. Tang et al. [28] conducted a study on neoadjuvant apatinib combined with oxaliplatin and capecitabine in the treatment of individuals with locally advanced adenocarcinoma of the stomach or gastroesophageal junction, and the results also showed good efficacy and controllable security. A number of studies [29–31] on neoadjuvant apatinib plus ICI combined with chemotherapy in the treatment of locally advanced GC have been conducted or completed, and the results also showed a satisfactory clinical response, pathological response, and safety.

COX regression analysis results showed that the peritoneal metastasis (*P* < 0.001), XELOX regimen (*P* = 0.0291), chemotherapy cycle ≥ 6 cycles (*P* < 0.001), and combined treatment group (*P* < 0.001) are independent prognostic factors affecting PFS (Table 3). These results also indicate the efficacy of apatinib in combination with chemotherapy with or without immunotherapy in the first-line treatment of advanced GC.

Apatinib has achieved valuable therapeutic effects in various carcinomas, including gastric cancer [32]. Our work showed that AEs occurred in both two groups. Basically, consistent with previous phase II and phase III clinical studies of apatinib, common AEs mainly include leukopenia, neutropenia, thrombocytopenia, loss of appetite, vomiting, hypertension, proteinuria, and hand-foot skin reaction. There were no serious (Grade IV) AEs in both two groups. Most patients can be reversed through dose adjustment or symptomatic treatment, and no discontinuation due to AEs.

Our results show that apatinib in combination with chemotherapy with or without immunotherapy is safety and efficacy for first-line therapy of advanced GC patients. However, our study also has certain limitations. First, due to the limited number of individuals included in this work, no meaningful results can be obtained on the efficacy of apatinib combined with chemotherapy alone or apatinib plus ICI combined with chemotherapy in the treatment of advanced GC patients. Secondly, because this is a single-center retrospective study with a small sample, there is certain selection and reporting bias. Third, we did not evaluate the PD-1/PD-L1 expression levels in individuals treated with ICI and were unable to further explore the correlation between immunotherapy and patient genomes. Fourth, this research has not yet met the OS endpoint. The specific survival advantage of apatinib in combination with chemotherapy with or without immunotherapy for first-line therapy of patients with advanced GC is still unclear. Prospective, multi-center, large-sample, and long-term follow-up cohort studies are still needed.

In summary, compared with chemotherapy alone, apatinib in combination with chemotherapy with or without immunotherapy can substantially optimize the ORR and prolong PFS in first-line advanced GC patients with acceptable tolerability. However, multicenter research is needed to explore the improvement of this treatment on the long-term prognosis of patients.

## Data Availability

Data, code, and other materials are available on request from the authors.
